# Feasibility of [^99m^Tc]Tc-Mannosylated Human Serum Albumin for Lymphoscintigraphy: A Phase 1/2 Clinical Trial

**DOI:** 10.1007/s13139-025-00951-z

**Published:** 2025-09-22

**Authors:** Hyunpil Sung, Minseok Suh, Hyun Gee Ryoo, Jin Chul Paeng, Wonshik Han, Jae-Min Jeong, Kweon Kim, Gi Jeong Cheon

**Affiliations:** 1 Department of Nuclear Medicine, Seoul National University Hospital, 101 Daehak-ro, Jongno-gu, Seoul, 03080 Republic of Korea; 2 Department of Molecular Medicine and Biopharmaceutical Sciences, Graduate School of Convergence Science and Technology, Seoul National University, Seoul, Republic of Korea; 3 Institute of Radiation Medicine, Seoul National University College of Medicine, Seoul, Republic of Korea; 4 Department of Surgery, Seoul National University Hospital, Seoul, Republic of Korea; 5 CellBion Co. Ltd, Seoul, Republic of Korea

**Keywords:** Sentinel lymph node, Lymphoscintigraphy, Mannosylated human serum albumin, Phase I clinical trial

## Abstract

**Purpose:**

Lymphoscintigraphy is crucial for diagnosis of lymphatic abnormality and sentinel lymph node (SLN) detection in surgery. We investigated the feasibility of [^99m^Tc]Tc-mannosylated human serum albumin (MSA) as a new imaging agent for lymphoscintigraphy.

**Methods:**

In the first part, three healthy volunteers were enrolled to evaluate the safety and basic efficacy of [^99m^Tc]Tc-MSA, using [^99m^Tc]Tc-phytate as a comparator. In the volunteers, the two radiopharmaceuticals were injected into the interphalangeal web of bilateral feet on different days. Whole-body scans were performed serially until 24 h after the injection. The biodistribution, radiation dosimetry, and safety were evaluated. In the second step, six breast cancer patients planned for operation were enrolled. SLN imaging was performed using [^99m^Tc]Tc-MSA two days before operation, and again on the day of operation using [^99m^Tc]Tc-phytate. The efficacy of SLN detection was evaluated using a visual grade system and compared between the two different radiopharmaceuticals.

**Results:**

[^99m^ Tc]Tc-MSA and [^99m^Tc]Tc-phytate show comparable biodistribution in evaluated organs and inguinal lymph node stations. [^99m^Tc]Tc-MSA showed relatively slower migration speed through lymphatic vessels and clearer visualization of lymphatics than [^99m^Tc]Tc-phytate. No abnormality was observed in safety parameters. In the radiation dosimetry, whole body dose at usual injection dose (74 MBq) was estimated to be 236 µSv. In SLN imaging in breast cancer patients, [^99m^Tc]Tc-MSA showed higher target-to-background ratio than [^99m^Tc]Tc-phytate, with clear visualization of SLN with both imaging agents.

**Conclusion:**

[^99m^Tc]Tc-MSA is a safe and effective imaging agent for lymphoscintigraphy. Particularly, [^99m^Tc]Tc-MSA can be a more effective radiopharmaceutical for SLN imaging than [^99m^Tc]Tc-phytate.

## Introduction

Lymphoscintigraphy is a very effective diagnostic imaging in various lymphatic diseases such as lymphedema and lymphatic leakage. From lymphoscintigraphy, information about lymphatic drainage pathway, retention or obliteration of lymphatid flow can be obtained. Additionally, detection of a sentinel lymph node (SLN) using lymphoscintigraphy is crucial to make a surgical plan in some cancers. Currently, SLN biopsy using lymphoscigraphy radiopharmaceuticals is one of standard methods for effective surgery in breast cancer [1–3]. Currently, several radiopharmaceuticals are available for lymphoscintigraphy, such as [^99m^Tc]Tc-sulfur colloid, [^99m^Tc]Tc-antimony sulfide colloid [4]– [5]. In SLN detection, the particle size of radiopharmaceuticals is the key feature [6].

In Korea, the only currently available radiopharmaceutical agent used for lymphoscintigraphy is [^99m^Tc]Tc-phytate. After injection, [^99m^Tc]Tc-phytate forms complexs with calcium to be colloid in vivo. The size of [^99m^Tc]Tc-phytate colloid varies, with a range of 5–1,000 nm [7]– [8], and thus, there is limitation in reproducibility of colloid size. Additionally, the migration speed of [^99m^Tc]Tc-phytate is relatively fast compared to other lymphoscintigraphy agent and not optimal for usual SLN imaging procedures.

Albumin-based agents have been reported as effective imaging tracers for lymphoscintigraphy [9–12]. Human serum albumin has uniform size of 6–8 nm [13], and is highly stable in vivo. Among the albumin-based agents, [^99m^Tc]Tc-mannosylated human serum albumin (MSA) is expected to be an optimal agent for SLN imaging, as it has uniform size prefered for lymphoscintigraphy [14]– [15], and it binds to the mannose receptors that are expressed on specific cell types in lymph nodes [16–19]. Thus, [^99m^Tc]Tc-MSA has been developed as an imaging agent for lymphoscintigraphy and SLN detection, which underwent a phase 1/2 clinical trial.

Here, we report results of the phase 1/2 clinical trial on the use of [^99m^Tc]Tc-MSA for lymphoscintigraphy. In the first part, safety and biodistribution of [^99m^Tc]Tc-MSA were assessed in lower-extremity lymphoscintigraphy of healthy volunteers. In the second step, the efficacy of [^99m^Tc]Tc-MSA for SLN imaging was compared with that of [^99m^Tc]Tc-phytate.

## Materials and Methods

### Subjects and Study Design

A prospective, single-center, phase 1/2 clinical trial was designed to evaluate the safety, radiation dosimetry, and efficacy of [^99m^Tc]Tc-MSA compared with [^99m^Tc]Tc-phytate.

In the first part of the study, healthy adult volunteers were recruited after a general condition assessment and past medical history review. The primary objective of this part was to evaluate the safety profile, biodistribution, and radiation dosimetry of [^99m^Tc]Tc-MSA. Each subject underwent lower-extremity lymphoscintigraphy with [^99m^Tc]Tc-MSA. Approximately seven days later, adverse events—including allergic reactions and injection site irritation—were monitored, and lymphoscintigraphy was repeated using [^99m^Tc]Tc-phytate as a comparator. Safety was assessed by monitoring adverse events and laboratory results, and radiation dosimetry was calculated based on whole-body scintigraphy data.

In the second part of the study, patients with breast cancer scheduled for SLN biopsy and breast surgery were enrolled. The primary objective was to assess the efficacy of SLN detection using [^99m^Tc]Tc-MSA compared to [^99m^Tc]Tc-phytate. Each patient underwent SLN lymphoscintigraphy with [^99m^Tc]Tc-MSA two days prior to surgery, followed by a second SLN lymphoscintigraphy with [^99m^Tc]Tc-phytate on the day of operation. The two sets of images were compared in terms of SLN detectability and image quality using a visual grading scale.

Adverse events were categorized and graded according to the Common Terminology Criteria for Adverse Events (CTCAE, version 4.03). Laboratory evaluations including urinalysis, complete blood count, liver function tests, renal function tests were performed before and after radiopharmaceutical administration.

The trial was registered not in an open public registry, but in a Korean Governmental registry (https://nedrug.mfds.go.kr/searchClinic). The study was approved by the Institutional Review Board of the Seoul National University Hospital (1902-134-1015). All procedures performed in this study were in accordance with the ethical standards of the institutional research committee and the Helsinki Declaration and its later amendments or comparable ethical standards.

### Preparations of [^99m^Tc]Tc-MSA and [^99m^Tc]Tc-phytate

The basic labeling processes have been reported previously [9]. MSA labeling kit vials were manufactured and provided by the sponsor (Cellbion Co., Ltd, Seoul, Korea). The vials were kept in a refrigerator and placed at room temperature for 5 min before labeling. The vial was mixed with 2 mL of free ^99m^Tc solution and then incubated for 10 min at room temperature. [^99m^Tc]Tc-MSA was filtered using a 0.2 μm syringe filter (Minisart^®^, Sartorius AG, Göttingen, Germany), and tested for radioactivity, radiochemical purity and pH. The injected [^99m^Tc]Tc-MSA was prepared with a volume of 0.1 mL containing 37 MBq.

[^99m^Tc]Tc-phytate was prepared using a commercial kit vial (Techne^®^ Phytate, PDRadiopharma Inc. Tokyo, Japan) according to the manufacturer’s instructions. The vial was mixed with 2 mL of free ^99m^Tc solution and then incubated for 10 min at room temperature as well. The labeled [^99m^Tc]Tc-phytate was tested for radioactivity, radiochemical purity and pH. [^99m^Tc]Tc-phytate was also prepared with a volume of 0.1 mL containing 37 MBq.

### Image Acquisition

In the healty subjects, [^99m^Tc]Tc-MSA was intradermally injected at the first and second interphalangeal webs (0.05 mL at each site) of both feet. Whole-body scans were performed at six time points; immediate post-injection (< 10 min), 20 min, 1 h, 2 h, 4 h, and 24 h after the injection. The whole-body scan were performed using the same dual-head gamma camera equipped with low-energy high-resolution collimator (Symbia EVO EXCEL, Simens Healthineers, Erlangen, Germany). The images were obtained using continuous bed motion (scan speed 10 cm/min). [^99m^Tc]Tc-phytate was injected at the same sites as [^99m^Tc]Tc-MSA. Whole-body scans were performed at three time points; immediate post-injection (< 10 min), 20 min, and 1 h, using the same protocol as [^99m^Tc]Tc-MSA.

In the breast cancer patients, [^99m^Tc]Tc-MSA and [^99m^Tc]Tc-phytate were intradermally injected at the subareolar area. Images were scheduled to be acquired at four time points; post-injection, 20 min, 1 h and 2 h after injection using the same gamma camera and protocol. The detector was located on anterior chest covering both breasts, and images were obtained for 5 min using a matrix size of 128 × 128. However, to minimize patient burden in this clinical setting, imaging in Part 2 was primarily focused on the post-injection and 1 h time points for comparative evaluation.

### Image Analysis for Radiation Dosimetry

A region-of-interest (ROI) was manually drawn for each organ on [^99m^Tc]Tc-MSA images of the healthy subject. On both the anterior and posterior images, ROIs were drawn for major organs (heart, liver, spleen, lungs, and bladder), injection sites, and whole body. Relative accumulation ratio was defined as the ratio of the count within the ROIs drawn on each organs to the total count of the whole body at the immediate post-injection scan, with correction for decay. Geometric means were calculated from the ROI counts, and the Riemann integration method was applied to obtain the time-integrated activity. For organ dosimetry, the ‘total disintegration per unit activity administered (residence time)’ was calculated by dividing the time-integrated activity by the injected activity.

The organ doses of standard individuals were calculated using OLINDA/EXM (Vanderbilt University, TN, USA), with the adult phantom options. The heart, liver, spleen, lungs were selected as the major source organs. From the decay-corrected whole body and bladder counts, urinary excretion fraction and biological half-life were calculated and the dynamic bladder model was applied with assuming voiding frequency of 4 h. The radioactivity in the injection site and the local radiation dose were ignored from the dosimetry according to a previous report [20].

### Efficacy of Lymph Node Detection

In the healthy subjects, the characteristics of lymphatic migration and the detection sensitivity for lymphatics and LNs were qualitatively compared between [^99m^Tc]Tc-phytate and [^99m^Tc]Tc-MSA scans.

In the breast cancer patients, axillary SLN uptake on [^99m^Tc]Tc-phytate and [^99m^Tc]Tc-MSA scans was visually scored by consensus of two experienced nuclear medicine physicians using a 4-grade system; 1 (no), 2 (discernible but mild), 3 (moderate), 4 (intense). Afterward, a circular ROI was drawn to encompass the axillary SLN and another circular ROI (size 40 ± 5 cm^2^) was drawn on the contralateral chest to measure the mean count. Target-to-background ratio (TBR) was calculated as the maximal count of axillary SLN ROI divided by the mean count of the chest ROI. The TBRs of [^99m^Tc]Tc-phytate and [^99m^Tc]Tc-MSA scans were compared.

### Statistical Analysis

For the comparison of paired data, the Wilcoxon signed rank test was used. Statistical analysis were performed using R studio (version: 4.3.3). *P*-values < 0.05 were considered significant.

## Results

### Subjects and Safety

In the first part, three healthy volunteers completed the trial protocol. The volunteers were a 21-year-old female, a 28-year-old female and a 38-year-old male. All of the three volunteers had no notable medical history. No adverse events were observed in any of the participants, based on CTCAE criteria, and no clinically significant abnormalities were detected in urinalysis, complete blood count, liver function tests, or renal function tests. The laboratory test results of the participants are shown in Table 1.

**Table 1:** Laboratory test results of patients

	Baseline	Follow up	Reference
Hemoglobin (g/dL)	14.9 ± 2.1	14.4 ± 2.1	13–17
Hematocrit (%)	42.4 ± 6.6	41.9 ± 7.0	39–52
RBC count (×10^6^/µL)	5.00 ± 0.42	4.93 ± 0.51	4.2–6.3
Platelet count (×10^6^/µL)	275 ± 48	268 ± 46	130–400
WBC count (×10^6^/µL)	8.32 ± 3.00	5.66 ± 1.50	4–10
Basophil (%)	0.5 ± 0.1	0.6 ± 0.3	0–2
Eosinophil (%)	1.3 ± 0.9	2.2 ± 1.0	1–5
Neutrophil (%)	69.0 ± 9.3	61.0 ± 8.2	50–70
Lymphocyte (%)	23.6 ± 9.7	29.6 ± 9.2	20–44
Monocyte (%)	5.7 ± 0.4	6.7 ± 1.7	2–9
ALT (IU/L)	26 ± 23	25 ± 19	1–40
AST (IU/L)	21 ± 9	21 ± 9	1–40
LDH (IU/L)	158 ± 22	159 ± 1	100–225
CPK (mg/dL)	101 ± 79	119 ± 102	20–270
BUN (mg/dL)	11.3 ± 2.5	13.0 ± 2.0	10–26
Creatinine (mg/dL)	0.79 ± 0.03	0.75 ± 0.05	0.7–1.4
Fasting glucose (mg/dL)	94 ± 12	90 ± 17	70–110
Urinalysis			
pH	6.4 ± 1.0	5.8 ± 0.6	5.5–7
Specific gravity	1	1	1
Protein	1	1	1
Glucose	1	1	1
WBC	1	1	1
RBC	1	1	1
Ketone	1	1	1

In the second part, six patients with breast cancer completed the trial protocol (age 50.0 ± 4.7 y, all female). Five patients had invasive ductal carcinoma, and one had invasive lobular carcinoma. The pathologic staging was IA in 5 patients, and IIA in 1 patient. Four patients had cancers in right breast, while two patients had cancers in left breast. All participants tolerated the scan procedures without any drug-related adverse events.

### Image Characteristics and Radiation Dosimetry in Healthy Volunteers

**Fig. 1:**
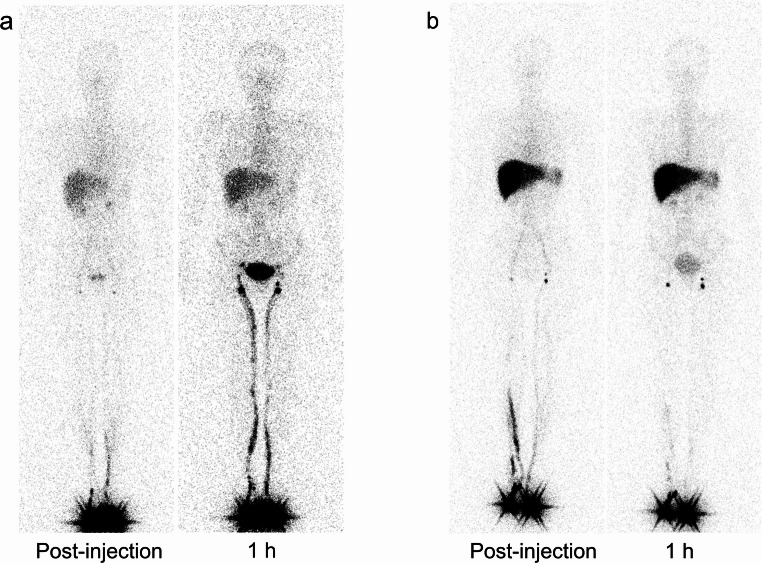
Representitive whole body scans at immediate and 1 h post-injection in normal volunteers. (**a**) [^99m^Tc]Tc-MSA (**b**) [^99m^Tc]Tc-phytate. [^99m^Tc]Tc-MSA shows more prominent bilateral inguinal lymph node activity compared to [^99m^Tc]Tc-phytate. However, [^99m^Tc]Tc-MSA demonstrates slower migration to the liver, as evidenced by lower hepatic uptake at both time points

**Fig. 2:**
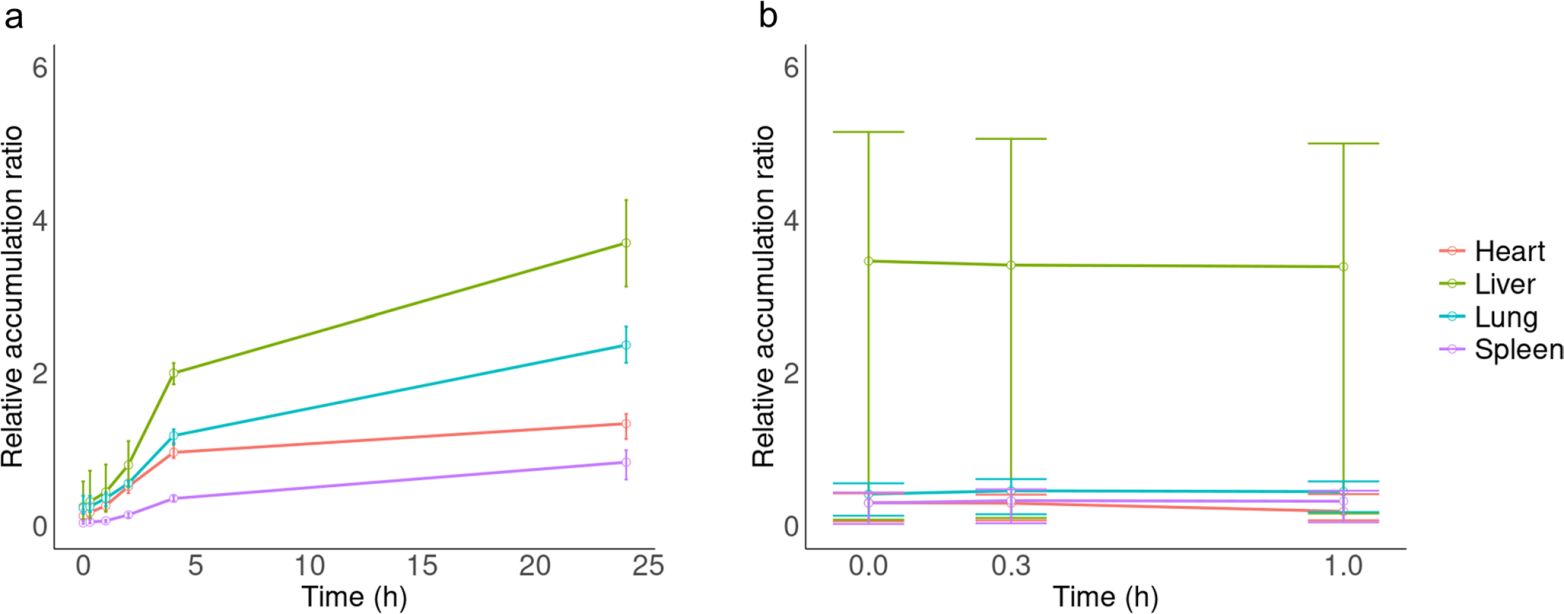
Time course of relative accumulation ratio for (**a**) [^99m^Tc]Tc-MSA and (**b**) [^99m^Tc]Tc-phytate. Each dot represents the median value at each time point, and the error bars indicate the range of values observed across the subjects

Representative images of whole body scans at post-injection and 1 h time points are shown in Fig. 1. Compared to [^99m^Tc]Tc-phytate, [^99m^Tc]Tc-MSA exhibited slower migration through the lymphatic vessels and a more distinct pattern of accumulation in the lymph nodes. Particulary, lymphatic duct was more clearly visualized on [^99m^Tc]Tc-MSA scan than [^99m^Tc]Tc-phytate scan. The mean relative accumulation ratios of bilateral inguinal lymph nodes for [^99m^Tc]Tc-MSA ranged from 0.005 to 0.011% at post-injection and from 0.451 to 1.782% at 1 h. For [^99m^Tc]Tc-phytate, the relative accumulation ratios ranged from 0.005 to 0.022% at post-injection and from 0.050 to 0.710% at 1 h. Time-courses of organ uptake are shown in Fig. 2. The accumulation of [^99m^Tc]Tc-MSA showed gradual increase until 24 h in all evaluated organs, particularly in the liver. The activities in the lungs and heart also showed increase, probably due to blood pool activity.

**Fig. 3:**
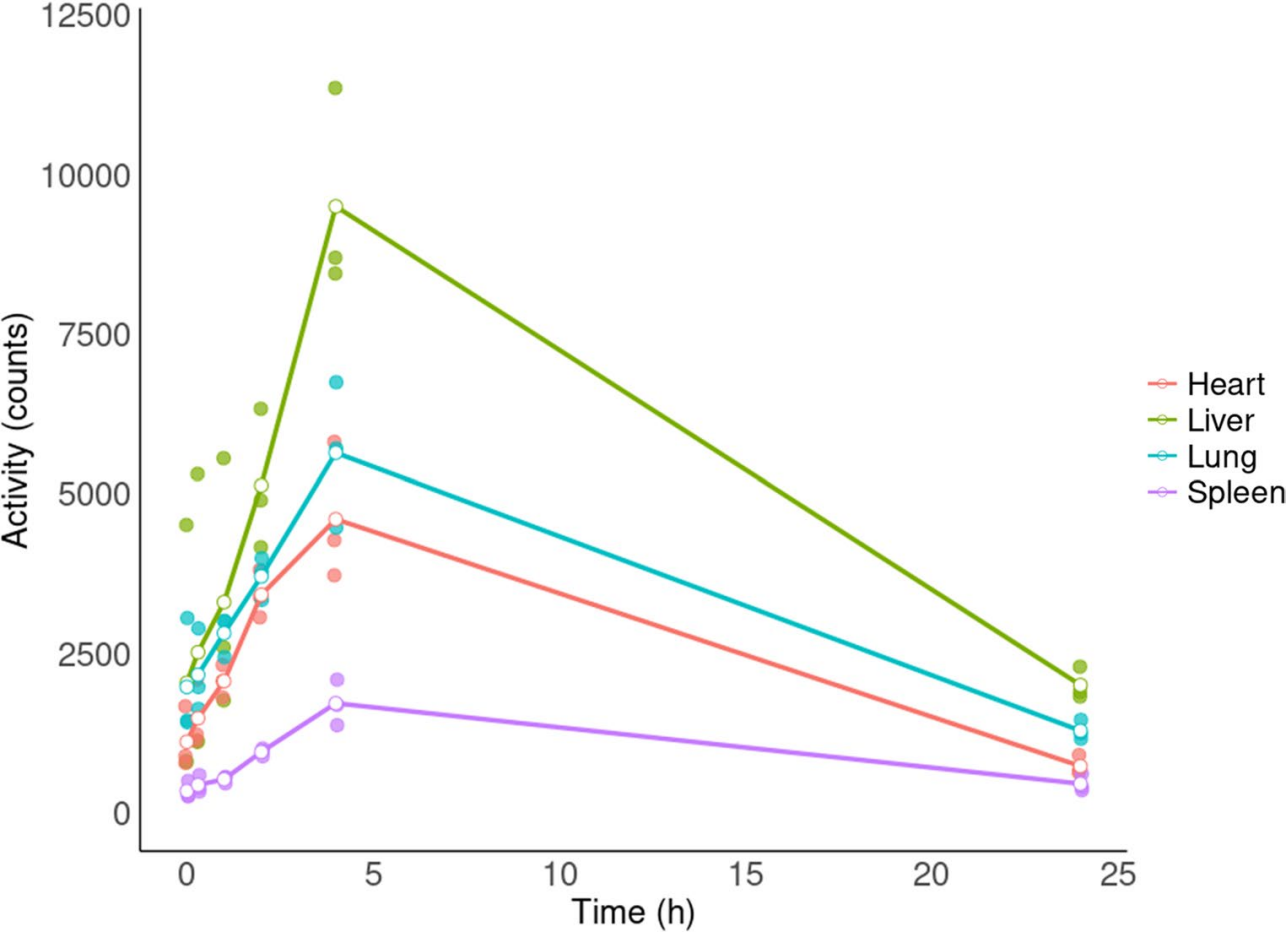
Time-activity curve of [^99m^Tc]Tc-MSA for major organs. Dots represent the activity measured in each organ at each time point. White dots indicate the mean activity across subjects at each time point

Time-activity curves of the major organs for radiation dosimetry are shown in Fig. 3. The calculated effective dose of whole body at usual injection dose was 235.6 ± 29.6 µSv/74 MBq. Urinary bladder was the organ of the highest effective dose, followed by ovaries and lungs. The calculated radiation dose of each organ is shown in Table 2.

**Table 2:** Radiation dosimetry for the adult Phantom using the data from healthy subjects. LLI; lower largeiintestine. ULI; upper large intestine

Organ	Effective dose contribution (µSv/MBq)	Effective dose contribution at usual dose (µSv/74 MBq)
Adrenals	0.011 ± 0.001	0.8 ± 0.1
Brain	0.006 ± 0.001	0.5 ± 0.1
Breasts	0.061 ± 0.008	4.5 ± 0.6
Gallbladder Wall	0.000 ± 0.000	0.0 ± 0.0
LLI Wall	0.343 ± 0.051	25.4 ± 3.8
Small Intestine	0.012 ± 0.002	0.9 ± 0.1
Stomach Wall	0.232 ± 0.030	17.2 ± 2.2
ULI Wall	0.011 ± 0.002	0.8 ± 0.1
Heart Wall	0.000 ± 0.000	0.0 ± 0.0
Kidneys	0.009 ± 0.001	0.7 ± 0.1
Liver	0.150 ± 0.005	11.1 ± 0.4
Lungs	0.318 ± 0.018	23.5 ± 1.4
Muscle	0.009 ± 0.001	0.6 ± 0.1
Ovaries	0.578 ± 0.086	42.8 ± 6.4
Pancreas	0.012 ± 0.001	0.9 ± 0.1
Red Marrow	0.200 ± 0.028	14.8 ± 2.1
Osteogenic Cells	0.044 ± 0.007	3.3 ± 0.5
Skin	0.011 ± 0.002	0.8 ± 0.1
Spleen	0.020 ± 0.001	1.5 ± 0.1
Testes	0.000 ± 0.000	0.0 ± 0.0
Thymus	0.009 ± 0.001	0.7 ± 0.1
Thyroid	0.080 ± 0.012	5.9 ± 0.9
Urinary Bladder Wall	1.048 ± 0.150	77.6 ± 11.1
Uterus	0.021 ± 0.003	1.5 ± 0.2
Remainder of body	0.000 ± 0.000	0.0 ± 0.0
Whole body	3.184 ± 0.400	235.6 ± 29.6

### Efficacy of Lymphatics and Lymph Node Detection in Breast Cancer Patients

**Fig. 4:**
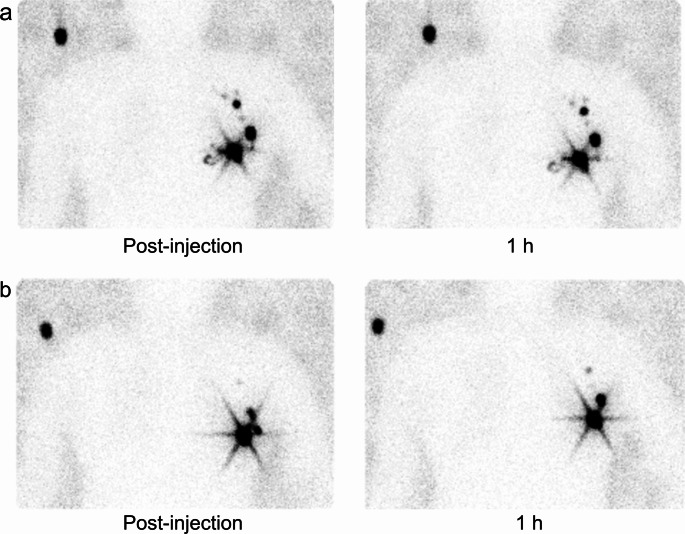
Representitive images at immediate and 1 h post-injection in a patient with left breast cancer. (**a**) [^99m^Tc]Tc-MSA (**b**) [^99m^Tc]Tc-phytate. Intense activity is observed at the injection site, and the sentinel lymph node is clearly visualized in both radiopharmaceuticals

**Fig. 5:**
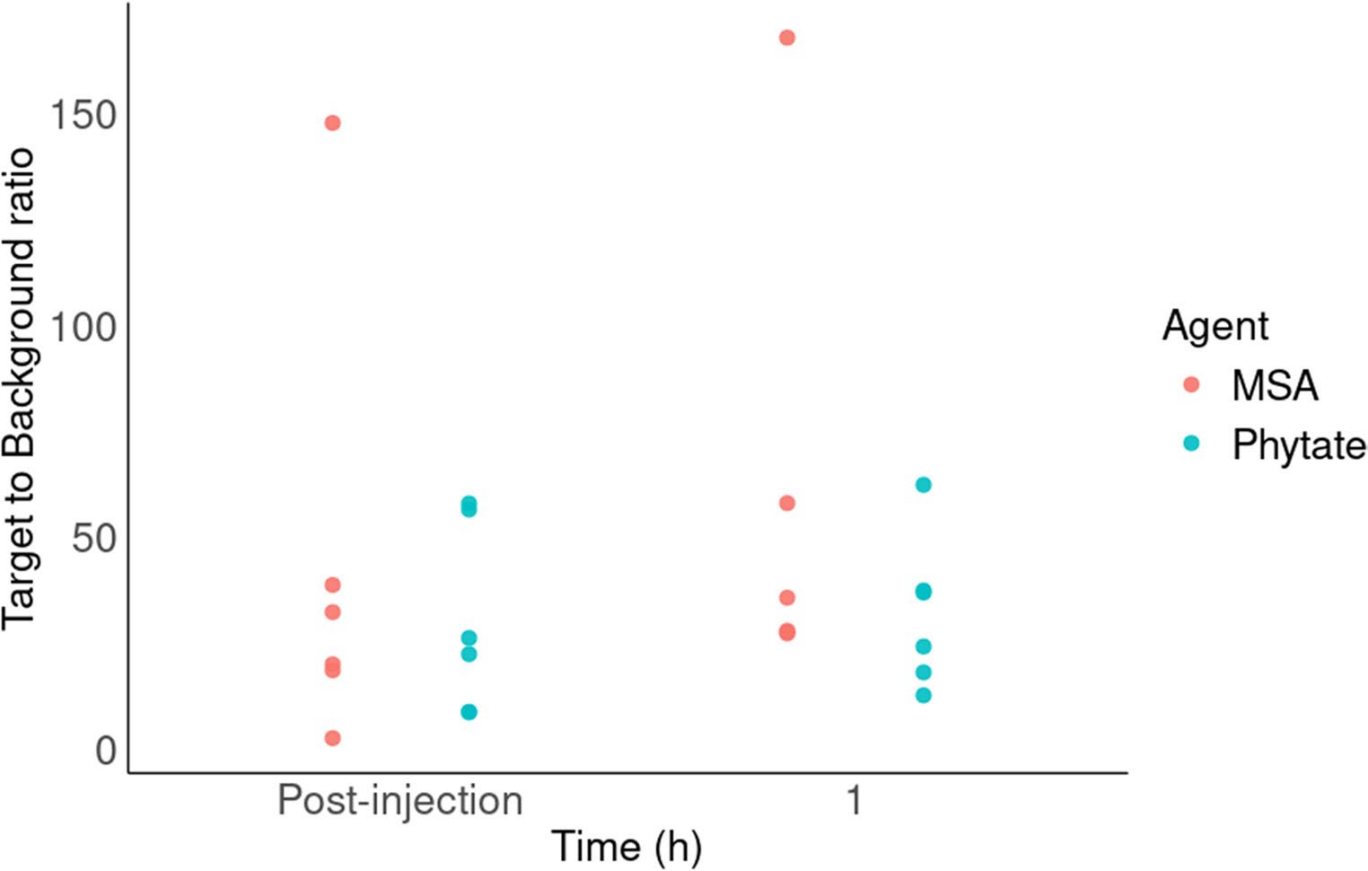
Target-to-background ratio of [^99m^Tc]Tc-MSA and [^99m^Tc]Tc-phytate at immediate and 1 h post-injection in SLN imaging. Each dot represents the target-to-background ratio of an individual patient for each radiopharmaceutical. For [^99m^Tc]Tc-MSA at 1 h timepoint, some dots overlap due to identical or similar target-to-background ratio values

Representative images of SLN imaging in breast cancer patients at post-injection and 1 h time points are shown in Fig. 4. Sentinel lymph nodes were well visualized on both [^99m^Tc]Tc-MSA and [^99m^Tc]Tc-phytate. The visual grades of SLN uptake are presented in Table 3. All sentinel lymph nodes were visualized as grade 4 in all patients. The TBRs of both agents at immediate post-injection and 1 h are shown in Fig. 5. TBR of [^99m^Tc]Tc-MSA at 1 h (median 31.9, range 27.4–167.4) was slightly higher than that of [^99m^Tc]Tc-phytate (median 30.6, range 12.8–62.4) despite large variances. However the difference was not statistically significant (post-injection; p-value = 1, 1 h; p-value = 0.437).

**Table 3:** Visual grade of uptake in Sentinel lymph nodes

	Post-injection		20 min		1 h		2 h	
Patients	Phytate	MSA	Phytate	MSA	Phytate	MSA	Phytate	MSA
1	4	4	N/D^*^	N/D	4	4	N/D	4
2	4	4	N/D	N/D	4	4	N/D	4
3	4	4	N/D	N/D	4	4	N/D	4
4	4	4	N/D	N/D	4	4	N/D	4
5	4	4	4	N/D	4	4	N/D	4
6	4	4	4	N/D	4	4	N/D	4

*: Not done

## Discussion

Both [^99m^Tc]Tc-MSA and [^99m^Tc]Tc-phytate were well tolerated without any drug-related adverse event in healthy participants and patients with breast cancer. Both [^99m^Tc]Tc-MSA and [^99m^Tc]Tc-phytate migrated along the lymphatics and well visualized lymph nodes clearly. At 1 h time point, the two radiopharmaceuticals exhibited different migration speed through the lymphatic vessels and pattern of accumulation in the lymph nodes, although both of them are acceptable in clinical practice. In breast cancer patients, [^99m^Tc]Tc-MSA images effectively visualized SLNs on the injected side of breasts, demonstrating comparable performance to [^99m^Tc]Tc-phytate. Additionally, [^99m^Tc]Tc-MSA images showed a slightly higher TBR than [^99m^Tc]Tc-phytate in this small cohort, suggesting its potential clinical utility for SLN visualization which warrants further investigation in larger-scale studies.

Lymphoscintigraphy is employed in clinical practice for the diagnosis of lymphedema or lymphatic leakage, and for the assessment of therapeutic outcomes of treatments for such conditions [21]. In addition to lymphedema management, lymphoscintigraphy is an essential tool in SLN mapping, which has become a cornerstone in the staging and treatment planning of breast cancer [1–3]. In Korea, the only currently available radiopharmaceutical agent used for lymphoscintigraphy is [^99m^Tc]Tc-phytate. Upon injection, it forms colloids that exhibit a broad particle size distribution. It has been noted to have limitations in reproducibility [7]– [8]. Given these limitations, there is a growing need for novel radiopharmaceutical agents that offer improved size consistency, slower lymphatic migration and enhanced imaging performance optimized for SLN detection.

[^99m^Tc]Tc-MSA was reported to bind to the mannose receptor on the macrophage surface, which may result in prolonged retention within lymph nodes [16–19]. The characteristics may be beneficial in SLN detection, especially when there is an extended time interval between radiopharmaceutical injection and surgical procedure. Most of the injected [^99m^Tc]Tc-MSA particles have a uniform size [14]– [15]. According to previous study, the particle size of [^99m^Tc]Tc-MSA was measured at 8.534 ± 1.224 nm using dynamic light scattering [22]. In comparision, only 8–10% of [^99m^Tc]Tc-phytate was colloidal, the size of which was approximately 8 nm [7]. Thus, the lymphoscintigraphy using [^99m^Tc]Tc-phytate is vulnerable to inconsistency. Considering the differences between [^99m^Tc]Tc-MSA and [^99m^Tc]Tc-phytate, the characteristics of [^99m^Tc]Tc-MSA enhances the targeting of SLN and improves the accuracy of SLN imaging, as it facilitates more efficient and focused uptake in the LNs.

In the context of radiation safety, [^99m^Tc]Tc-MSA was assessed through hematologic, hepatic and renal function tests, without significant issues in any of the subjects. While there remains a potential for adverse effects associated with the radiopharmaceutical itself, but it is considered minimal [23]. Also in the radiation dosimetry, the radiation delivered to a patient was approximately 0.24 mSv. Given that ^99m^Tc-labeled agents including [^99m^Tc]Tc-phytate commonly used in lymphoscintigraphy exhibit patient effective dose ranging 0.15–2.2 mSv/74MBq [24], this falls within the lower range and is clinically acceptable. It is anticipated that the radiation retained in the lymph nodes would have negligible impact on pathologists or medical technician [25]– [26]. Although assessing the radiation dose form residual radioactivity at the injection site may be challenging, it is unlikely to present a major concern.

The present exploratory study suggests the potential of [^99m^Tc]Tc-MSA as a promising radiopharmaceutical agent for lymphoscintigraphy and SLN detection. [^99m^Tc]Tc-MSA may offer improved quality compared to [^99m^Tc]Tc-phytate for the evaluation of lymphatic disorders and surgical treatment planning in cancer patients. However, further validation in larger, multicenter trials is required to confirm its clinical impact. Currently, radopharmaceuticals that are clinically available for lymphoscintigraphy is limited, [^99m^Tc]Tc-MSA can be a new option, if it is approved by the authority. In Korea, [^99m^Tc]Tc-phytate is approved for both hepatic/splenic scintigraphy and lymphoscintigraphy, and has long been used as the standard agent for lymphatic imaging. However, its primary indication also includes hepatic and splenic applications, which should be considered when interpreting its performance in lymphatic imaging.

There are some limitations in the present study. First, the numbers of enrolled volunteers and patients were small, which may affect the generalizability of the findings. However, considering that the principles behind lymphoscintigraphy are relatively straightforward, it is unlikely that the key results would be altered by a larger sample size. Larger, multicenter, phase 3 clinical studies are needed to further validate the safety and clinical efficacy of [^99m^Tc]Tc-MSA. Second, the long-term safety and potential adverse effects of [^99m^Tc]Tc-MSA was not evaluated in this study. However, it can be presumed that this is not a critical problem in diagnostic radiopharmaceuticals, as they are used in extremely low amounts and exhibit little pharmacologic effects. Furthermore, as [^99m^Tc]Tc-MSA is an albumin-based agent with high biocompatibility and predominant hepatic metabolism, the likelihood of long-term adverse effects is considered minimal.

## Conclusion

[^99m^Tc]Tc-MSA was well tolerated without drug-related adverse events and demonstrated compatible result to [^99m^Tc]Tc-phytate for lymphoscintigraphy study and SLN visualization in this exploratory study. Additionally, the radiation dose is considered to be within an acceptable and appropriate level. This findings suggest that [^99m^Tc]Tc-MSA may be a saft and efficient option for lymphoscintigraphy study and SLN detection, but further validation in larger clinical studies is warranted.

## Data Availability

Contact there corresponding author for data requests.
